# Multiple Inositol Polyphosphate Phosphatase Compartmentalization Separates Inositol Phosphate Metabolism from Inositol Lipid Signaling

**DOI:** 10.3390/biom13060885

**Published:** 2023-05-24

**Authors:** Jia Yu, Barbara Leibiger, Shao-Nian Yang, Stephen B. Shears, Ingo B. Leibiger, Per-Olof Berggren, Christopher J. Barker

**Affiliations:** 1 The Rolf Luft Research Center for Diabetes and Endocrinology, Karolinska Institutet, Karolinska University Hospital, SE-171 76 Stockholm, Sweden; 2 Inositol Signaling Section, NIEHS, 111, Alexander Drive, Research Triangle Park, Durham, NC 27709, USA

**Keywords:** multiple inositol polyphosphate phosphatase (MINPP1), inositol phosphate, inositol lipid, phosphatidylinositol 3,4,5-trisphosphate, PIP_3_, pancreatic beta cell

## Abstract

Multiple inositol polyphosphate phosphatase (MINPP1) is an enigmatic enzyme that is responsible for the metabolism of inositol hexakisphosphate (Ins*P*_6_) and inositol 1,3,4,5,6 pentakisphosphate (Ins(1,3,4,5,6)*P*_5_ in mammalian cells, despite being restricted to the confines of the ER. The reason for this compartmentalization is unclear. In our previous studies in the insulin-secreting HIT cell line, we expressed MINPP1 in the cytosol to artificially reduce the concentration of these higher inositol phosphates. Undocumented at the time, we noted cytosolic MINPP1 expression reduced cell growth. We were struck by the similarities in substrate preference between a number of different enzymes that are able to metabolize both inositol phosphates and lipids, notably IPMK and PTEN. MINPP1 was first characterized as a phosphatase that could remove the 3-phosphate from inositol 1,3,4,5-tetrakisphosphate (Ins(1,3,4,5)*P*_4_)_._ This molecule shares strong structural homology with the major product of the growth-promoting Phosphatidyl 3-kinase (PI3K), phosphatidylinositol 3,4,5-trisphosphate (PtdIns(3,4,5)*P*_3_) and PTEN can degrade both this lipid and Ins(1,3,4,5)*P*_4_. Because of this similar substrate preference, we postulated that the cytosolic version of MINPP1 (cyt-MINPP1) may not only attack inositol polyphosphates but also PtdIns(3,4,5)*P*_3_, a key signal in mitogenesis. Our experiments show that expression of cyt-MINPP1 in HIT cells lowers the concentration of PtdIns(3,4,5)*P*_3_. We conclude this reflects a direct effect of MINPP1 upon the lipid because cyt-MINPP1 actively dephosphorylates synthetic, di(C4:0)PtdIns(3,4,5)*P*_3_ in vitro. These data illustrate the importance of MINPP1′s confinement to the ER whereby important aspects of inositol phosphate metabolism and inositol lipid signaling can be separately regulated and give one important clarification for MINPP1′s ER seclusion.

## 1. Introduction

Inositol polyphosphates are involved in key signaling processes in cell regulation, such as Ca^2+^ signaling, membrane trafficking, regulation of serine-threonine phosphatases, gene transcription, nuclear mRNA transport, nucleolar regulation, insulin secretion and signaling, phosphate regulation [1,2,3,4,5,6], DNA maintenance [7] and the control of cell growth [8], including apoptosis [9,10]. Inositol lipids are also involved in many of the same processes [11,12,13,14] and others, including cytoskeletal regulation [15].

Much interest has been focused on the products of phosphatidylinositol 3-OH kinases (PI3Ks) and their downstream targets [11,13]. PI3Ks are predominantly, but not exclusively, activated by mitogens [11,13]. It has emerged that there is some degree of cross-talk between these previously divergent inositol phosphate and lipid pathways. Products of higher inositol polyphosphate metabolism can interact with PI3K pathways at several levels [10,16,17,18]. Specifically, PTEN, an established tumor suppressor with 3-phosphatase activity towards phosphatidylinositol 3,4,5-trisphosphate (PtdIns(3,4,5)*P*_3_) [19], has been shown to have Ins(1,3,4,5,6)*P*_5_ as an additional substrate [17]. Ins(1,3,4,5,6)*P*_5_ has been shown to antagonize the action of PtdIns(3,4,5)*P*_3_ by blocking the activation of Akt [10], as has 5-PP-Ins*P*_5_, also known as IP_7_ [18]. 

Multiple inositol polyphosphate phosphatase (MIPP/MINPP1) dephosphorylates inositol polyphosphates Ins(1,3,4,5,6)*P*_5_ and Ins*P*_6_ to a number of lower inositol polyphosphates [17,20,21], including Ins(1,4,5)*P*_3_ [22,23]. It has been implicated in the regulation of bone and cartilage formation [24], ER stress and apoptosis [25] and most recently, a human genetic disorder, pontocerebellar hypoplasia, has been associated with a loss of function mutation in MINPP1 [26]. This disease is accompanied by a profound impact on cognitive function and life expectancy [26] and thus brings new importance to this enigmatic enzyme. 

Full-length MINPP1 has an N-terminal ER targeting sequence [21], and in rat liver cells and mouse embryonic fibroblasts, most endogenous MINPP1 is restricted to the ER lumen [27,28]. In 3T3 cells, ectopically-expressed MINPP1 is also concentrated in ER [28]. Nevertheless, overexpression of full-length MINPP1 does reduce cellular levels of Ins(1,3,4,5,6)*P*_5_ and Ins*P*_6_ [28], and very recent work suggests these are also targets of the endogenously expressed enzyme [29]. Therefore, it is generally anticipated that inositol phosphate substrates must enter the ER. Or, under appropriate circumstances, MINPP1 may exit the ER. There is currently no explanation for this apparently circuitous method of regulating inositol polyphosphates normally resident in the cytosolic compartment. It is likely that the degradation of higher inositol polyphosphates to lower forms has some still-unknown purpose in the ER compartment, and/or the restriction of MINPP1 in the ER prevents unwanted metabolic events in the cytosol. In regard to the latter suggestion, the literature demonstrates a number of examples in which enzymes that metabolize inositol lipids can also metabolize inositol phosphates, including PTEN. Interestingly, although PTEN and MINPP1 have no sequence homology, even at their active sites, both proteins can dephosphorylate Ins(1,3,4,5)*P*_4_ and Ins(1,3,4,5,6)*P*_5_ in vitro at least [19,21]. As a model for studying the consequences for cells in which MINPP1 is no longer constrained to the ER, a truncated phosphatase lacking its ER-targeting sequence has been expressed in 3T3 cells [28]. In these experiments, MINPP1 inhibited cell growth [28]. This growth inhibition was previously ascribed to the degradation of the higher inositol polyphosphates, which are its principal substrates [20,21]. In this study with the insulin-secreting HIT cells as a model, we report that cyt-MINPP1 inhibits cell growth because the enzyme hydrolyzes PtdIns(3,4,5)*P*_3_. Thus, the ER confinement of MINPP1 allows the separation of inositol polyphosphate metabolism from the regulation of an important inositol lipid signal and explains, in part, the necessity of MINPP1′s ER location.

## 2. Materials & Methods

### 2.1. Preparation of GFP-Linked MINPP1 Constructs

Plasmids: Construction of pCI.cyt-MINPP1 and pCI.GFP-cyt-MINPP1 was described elsewhere [23]. Encoded by plasmid pCI.hr MINPP1 is a chimera made up of the 1st 39 amino acids of human MINPP1 (this includes the signal peptide, that is, amino acids 1-30). This is fused to the rat MINPP1 sequence, which is identical to that of cyt-MINPP1 without the FLAG tag. For the generation of pCI.hrMINPP1, we initially introduced a SalI site into the cDNA of human MINPP1. Codons were exchanged for amino acids V39A40 from GTG GCC to GTC GAC. We then introduced a SalI site into the spacer linking the DNA sequence encoding the N-terminal FLAG-tag and that encoding rat MINPP1. This was achieved by exchanging nucleotides GGC GCC versus GTC GAC in pCI.cyt-MINPP1. Lastly, we swapped the DNA sequences encoding the FLAG-tag in pCI.cyt-MINPP1 by the DNA sequence that encodes the first 39 amino acids of human MINPP1, combining both sequences in-frame using the created SalI sites. To generate pCI.GFP-hrMINPP1, we subcloned the DNA sequence encoding the amino acids 1–39 of human MINPP1 in-frame in front of the GFP-r MINPP1 cDNA via SalI sites. The Quikchange Kit (Clontech/TakaRa, Göteborg, Sweden) was used to perform all nucleotide exchanges. The appropriate oligonucleotides were obtained from Proligo France SAS (Evry Courcouronnes, France). DNA sequence analysis was used to verify all vector constructions.

### 2.2. Cell Culture, Transfection and Labelling Protocols

HIT M2.2.2. cells were normally maintained in RPMI 1640 medium with 10% fetal bovine serum, glutamine and penicillin/streptomycin (Thermo Fisher Scientific/Life Technologies, Stockholm, Sweden) as described previously [30]. A modified RPMI 1640 medium, RPMI 1640-M, was used for experiments. This revised media included additional MgSO_4_ (to give a final concentration of 0.8 mM), 50 μM inositol and 5.5 mM glucose. The 10% serum in this medium had been dialyzed (1000 MW cut-off, Spectro Por, Thermofisher Scientific, Stockholm, Sweden) to remove the high inositol present in fetal serum.

HIT M2.2.2 cells were plated at about 10% of their confluent density into 35 or 92 mm dishes and grown in the RPMI-1640-M medium above for 24 h. The cells were then changed into the transfection medium, DMEM, with the same inositol concentration as the experimental RPMI-1640-M. Cyt-MINPP1 expression plasmid (pCI.FLAG- MINPP1) or vector alone were transfected into the cells using a Ca^2+^ phosphate precipitation technique [30]. The next day the cells were washed and reintroduced into the RPMI-1640-M medium and maintained for a further 24 h. To establish cell number and approximate volume, cells were trypsinized from the dish, counted and the volume estimated by measuring the diameter of spherical cells with calibrated graticule viewed under a microscope. An alternative method for determining cell number was used in the experiments involving green fluorescent protein (GFP)-tagged MINPP1 constructs. Fluorescent cells were identified using a laser scanning TCS-SP2 confocal microscope (Leica Microsystems CMS GmbH, Mannheim, Germany) and counted. 

For measurements of PtdIns(3,4,5)*P*_3_, cells were grown in 92 mm dishes with 20 μCi/mL [^3^H]*myo*-inositol for the 46 h period following transfection, which was sufficient to label the lipids to an apparent isotopic equilibrium [31].

### 2.3. Confocal Microscopy and Colocalization 

To confirm the GFP-tagged MINPP1 construct’s localization, confocal microscopy was carried out. Colocalization with the fluorescent ER marker Brefeldin A, BODIPY 558/568 conjugate (Molecular Probes) was determined. Laser scanning confocal microscopy was performed using the Leica TCS SP2 microscope above. This was equipped with a Leica HCX Pl Apo x63/1.20/0.17 UV objective lens as previously described [32]. The following settings were used: for GFP and Brefeldin A, BODIPY 558/568 conjugate fluorescence, excitation wavelength 488 nm (Ar laser) and 546 nm (HeNe laser), a 488/543 double dichroic mirror and detection at 505–525 nm for GFP and 565–595 nm for Brefeldin A, BODIPY 558/568 conjugate.

### 2.4. Inositol Lipid Analysis

For the determination of PtdIns(3,4,5)*P*_3_, an established quantitative lipid extraction protocol was used [31]. Only siliconized glass or plasticware and pipette tips were used during the extraction. Media was removed from the cells, and 1 mL of ice-cold 1 M HCl was added. A 10 µL aliquot of a Sigma Phosphoinositide mix (25 mg/mL) was immediately added as a lipid carrier, and the plate was left on ice for 20 min. Cells were then removed by a cell scraper, and the plates were washed with 2.73 mL of a solution comprising 0.6 mL of 1 M HCl, 5 mM of tetrabutyl ammonium sulphate, and 2.13 mL of methanol. The mixture was then split into 2 phases by the addition of chloroform (4.27 mL). The mixture was vortexed, and the phases were divided by centrifugation. The lower phase contained the inositol lipids and was added to tubes containing 1.43 mL of synthetic upper phase. The phases were mixed and centrifuged, and the lower phase was removed into clean tubes. Both the initial upper phase and the synthetic upper phase (left after removing the first synthetic lower phase wash) were sequentially re-extracted with 2.23 mL of synthetic lower phase, mixed and separated centrifugally. This last lower phase was combined with the originally washed lower phase, the tube filled with N_2_ and the lipid extract was stored at −20 °C. In order to determine PtdIns(3,4,5)*P*_3,_ the lipid extract was dried under N_2_, deacylated with methylamine exactly as described by Anderson et al., 1999 [31] and the products were stored at –20 °C until resolved by HPLC. 

### 2.5. HPLC

We performed high-performance liquid chromatography (HPLC) on a 25-cm Whatman Partisphere-SAX column (HiChrom/Avantor, Reading, UK). For the separation of deacylated inositol lipids, the gradient was generated from deionized H_2_O (buffer A) and 1.0 M (NH_4_)_2_ HPO_3_ adjusted to pH 3.8 with H_3_PO_4_ (buffer B). The gradient was as follows: 0 min, 0% B; 5 min, 0% B; 60 min, 15% B; 80 min, 15% B; 88 min, 20% B; 108 min, 30% B, 123 min, 100% B. To verify the identity of PtdIns(3,4,5)*P*_3_, we prepared a [^32^P]-labeled PtdIns(3,4,5)*P*_3_ standard by phosphorylating cold PIP_2_ (Sigma Aldrich, Stockholm, Sweden) and an equal proportion of phosphatidyl serine with recombinant PI3K (Alexis biochemicals, KELAB, Göteborg, Sweden) and [γ-^32^P]ATP (Perkin Elmer, Stockholm, Sweden)). The lipids were extracted, dried and deacylated and stored exactly as described above. Radioactivity was determined by the addition of Packard Ultima Flo AP scintillant and counting on a Packard CA 2000 scintillation counter (both from Perkin Elmer, Stockholm, Sweden).

### 2.6. Assay of cyt-MINPP1 Activity

Hydrolysis of PtdIns(3,4,5)*P*_3_ by soluble enzymes can be assayed using the naturally- occurring predominant form (which contains stearic acid (C18:0) in position sn-1 and arachidonic acid (C20:4) in position sn-2 [33] that is incorporated into membrane-like vesicles [34,35]. Others [36] have used phosphatidylserine-based vesicles seeded with synthetic di(C16:0) PtdIns(3,4,5)*P*_3_ (see [34,35]). Also recommended is a much simpler single-phase assay that utilizes synthetic short-chain fatty acids, e.g., di-(C4:0) PtdIns(3,4,5)*P*_3_ [34,35]. We took the latter approach to directly compare our rate of hydrolysis of PtdIns(3,4,5)*P*_3_ with the previously published rate of hydrolysis of InsP_6_ by MINNP1 [37]. In any case, in vivo, the fatty-acyl chains are buried in the lipid phase of the membrane and will not influence soluble MINPP1 activity.

Unless otherwise stated, assays were carried out exactly as described by Caffrey et al. [24]. Both a wild-type cyt-MINPP1 and a catalytically-compromised version were used (H370A). Assays were implemented in 70 µL of a buffer consisting of 50 mM HEPES pH 7.2/0.1 mg/mL BSA/50 µM di(C4:0)-PtdIns(3,4,5)*P*_3_ (Echelon Research labs, Salt Lake City, UT, USA). Reaction rates were indistinguishable at 25 and 100 µM, indicating assays were carried out under V_max_ conditions. The assay is not sensitive enough to determine Km. Assays were performed at 37 °C for 0, 15, 30 and 45 min and contained 0.027 µg of MINPP1. They were terminated by placing plates on ice. The Pi release was measured using a colorimetric assay in a microplate reader [38].

## 3. Results

### 3.1. Effect of cyt-MINPP1 on Cell Growth

**Figure 1:**
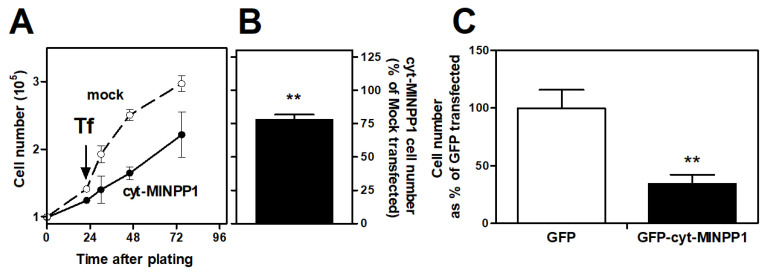
cyt-MINPP1 expression reduces cell growth. (**A**) Effect of cyt-MINPP1-expression on growth rate. Tf, time of transfection. Solid line, cyt-MINPP1 transfected cells, broken line, mock-transfected cells. One experiment performed in triplicate ± SEM is shown. One other experiment gave a similar result. (**B**) Numbers of cyt-MINPP1 expressing cells were measured at 48 h after transfection and expressed as a percentage of mock-transfected cells ± SEM from five separate experiments. (**C**) HIT M2.2.2 cells were expressing either GFP or GFP-cyt-MINPP1 and were imaged using confocal microscopy. Only cells that were fluorescent were incorporated into the subsequent analysis. The figure depicts the effect of GFP-tagged cyt-MINPP1 on the number of fluorescent cells as compared to GFP-expressing controls. Data are expressed as a percentage of the GFP control and are derived from three to four separate preparations. Data presented are mean ± SEM (** *p* < 0.01).

Cyt-MINPP1 expression significantly reduced cell growth compared to mock-transfected controls (Figure 1A,B) [28]. The reduction in cell growth, whilst significant, is not dramatic. However, the data is on the entire population of cells, and our transfection efficiency is only about 30%. Therefore, we are likely underestimating the impact of expressing MINPP1 in the cytosol. To overcome this issue, we made a cyt-MINPP1 GFP construct to further quantify the effect on cell growth.

Figure S1 illustrates that cyt-MINPP1 was evenly spread throughout the cytosol without specific colocalization with the ER marker, Brefeldin A- BODIPY 558/568 conjugate, which had a punctate pattern. We then used this construct to look at the effect on cell growth. Figure 1C shows that when only fluorescent, construct-expressing cells are considered, the differences in growth between GFP and GFP-cyt-MINPP1 expressing cells are considerably more marked. The number of GFP-cyt-MINPP1 expressing cells was significantly reduced by 70% compared to the control GFP-transfected cells. These data confirm the inhibitory effect of cyt-MINPP1 on the growth we saw in batch-transfected cells. 

### 3.2. Effect of cyt-MINPP1 Expression on PtdIns(3,4,5)P_3_

The physiological function of MINPP1 is generally recognized as an Ins(1,3,4,5,6)*P*_5_ and Ins*P*_6_ phosphatase, although the enzyme does show some 3-phosphatase activity towards Ins(1,3,4,5)*P*_4_ [20]. These observations led us to consider the possibility that PtdIns(3,4,5)*P*_3_ might also be a substrate for cyt-MINPP1. Since PtdIns(3,4,5)*P*_3_ is the product of type-1 phosphatidylinositol 3-kinase (PI3K), a group of enzymes intimately involved in the stimulation of cell growth [11,13], the degradation of PtdIns(3,4,5)*P*_3_ by cyt-MINPP1 could offer an explanation for the reduction in cell growth observed in Figure 1A–C.

**Figure 2:**
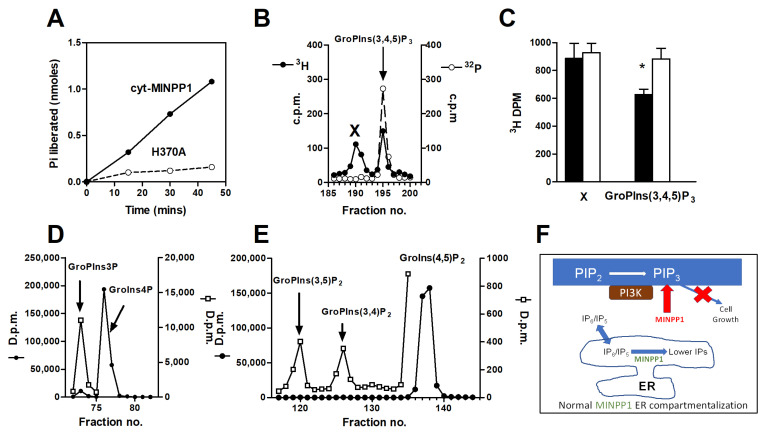
PtdIns(3,4,5)*P*_3_ is a substrate for purified cyt-MINPP1, and cyt-MINPP1 expression reduces the concentration of PtdIns(3,4,5)*P*_3_. (**A**) cyt-MINPP1 and a catalytically-compromised version were used (H370A) and incubated with C4-PtdIns(3,4,5)*P*_3_ for the times indicated. Activity was assessed by the release of Pi. The data are from a representative experiment where each time point was done in quintuplicate. Time courses were similar in two additional experiments. (**B**) In a deacylated lipid extract from cyt-MINPP1 transfected cells, two peaks are observed in the GroPIns(3,4,5)*P*_3_ region of the chromatogram: an unidentified peak X and a peak that co-eluted with an internal standard of [^32^P]GroPIns(3,4,5)*P*_3_. A similar extract from mock-transfected cells showed a similarly eluting GroPIns(3,4,5)*P*_3_, which also co-eluted with a [^32^P]-labeled standard. X is a side product of the deacylation of PtdIns(4,5)*P*_2_ [40]. From the original studies of Stephens et al. [39], it is likely to be Ins(2,4,5)*P*_3_. (**C**) Quantification of changes in peaks X and GroPIns(3,4,5)*P*_3_. Open bars mock-transfected and filled bars cyt- MINPP1 transfected. (Data ± SEM for one experiment carried out in triplicate are presented after normalization for the differences in cell number and volume, * *p* < 0.05, Students *t*-test. Two other experiments, also in triplicate, gave similar results. (**D**) Deacylation products of PtdIns3P and PtdIns4P, respectively. Note the large change of scale for GroPIns3P compared to GroPIns4P. (**E**) Deacylation products of PIP_2_ lipids; again, note the large change of scale for the 3-PI-based lipids. (**F**) Illustration of the compartmentalization of MINPP1 in the ER to separate inositol phosphate metabolism from inositol lipid signaling.

To test the above idea, we first assessed whether cyt-MINPP1 could dephosphorylate PtdIns(3,4,5)*P*_3_ in vitro. Figure 2A shows that di(C4:0)PtdIns(3,4,5)*P*_3_ is a substrate for cyt-MINPP1, whereas a catalytically dead mutant of cyt-MINPP1 (H370A) was ineffective. The estimated V_max_ for the reaction was 850 nmol/mg/min, which is considerably higher than that using either Ins(1,3,4,5,6)*P*_5_ (211 nmol/mg/min) [20], or Ins*P*_6_ (6.2 nmol/mg/min) as substrates [37].

To study the relevance of these observations in intact cells, we transfected HIT M2.2.2 cells with cyt-MINPP1, washed them and then incubated them with [^3^H] *myo*-inositol for 46 h. Subsequently, the inositol lipids were extracted and deacylated. PtdIns(3,4,5*)P*_3_ was identified in its deacylated form (GroPIns(3,4,5)*P*_3_) by the inclusion of an internal [^32^P]GroPIns(3,4,5)*P*_3_ standard in the HPLC run (Figure 2B). Levels of PtdIns(3,4,5)*P*_3_ were reduced by 26% by cyt-MINPP1 expression (Figure 2C). The data were corrected to account for the decreased cell number and the increased cell volume of the cyt-MINPP1-expressing cells. In the same experiments, there was no significant decrease in materials corresponding to PtdIns, PtdIns4*P* or PtdIns(4,5)*P*_2_, nor PtdIns(3,5)*P*_2_ and PtdIns(3,4)*P*_2_ (Figure S2A–C). There is a small reduction in PtdIns3*P,* which is likely to be due to the reduced level of PtdIns(3,4,5)*P*_3_ and its subsequent dephosphorylation route [39].

## 4. Discussion

The most important new finding in this study is that the levels of PtdIns(3,4,5*)P*_3_ are reduced in cyt-MINPP1 expressing cells, an observation that is matched by the ability of recombinant cyt-MINPP1 to degrade di(C4:0)-PtdIns(3,4,5*)P*_3_ in vitro. The agreement between these two independent sets of observations argues against the in-cell changes in PtdIns(3,4,5*)P*_3_ levels arising from the possible, but we believe unlikely, unknown, indirect effect of MINPP1. Our observations also indicate a greater substrate overlap than has previously been recognized. This observation also indicates that there is some overlap in the enzymological preferences between PTEN and MINPP1. What is also interesting is that the genes coding for these enzymes are both found at the same chromosomal locus [41], which is a hot spot for tumor suppressor genes [42]. The lower levels of PtdIns(3,4,5*)P*_3_ in cyt-MINPP1 overexpressing cells provide a satisfactory explanation for their slower rate of growth. It also, for the first time, rationalizes why MINPP1 is sequestered in the ER, namely to separate important inositol phosphate metabolism from a key mitogenic signaling pathway, that is, the phosphatidylinositol 3-OH kinase production of PtdIns(3,4,5)*P*_3_. The importance of this separation is underscored by a consideration of the enzyme kinetics. The apparent V_max_ with di(C4:0)-PtdIns(3,4,5*)P*_3_ as a substrate is 850 nmol/mg protein/min for MINPP1 compared to IP_6_ as a substrate with a V_max_ of 6.2 nmol/mg protein/min [37]. To put it in a wider perspective, the V_max_ of PTEN in vitro with di(C4:0)PtdIns(3,4,5*)P*_3_ as a substrate is 60 nmol/mg protein/min [17].

Earlier [28] and more recent works [26] clearly indicate that the control of cytosolic Ins*P*_6_ and Ins(1,3,4,5,6)*P*_5_ by ER-localized MINPP1 is both possible and important. A remaining open question is whether the products of their dephosphorylation by MINPP1 have some significant role in the ER. The recent characterization of MINPP1 metabolites [29] suggests that Ins(1,2,3)*P*_3_, a putative iron shuttle [43,44] and iron-specific antioxidant, [45] is a product of the endogenous, ER-confined MINPP1, as it is when this enzyme artificially expressed in the cytosol [23]. These recent studies [29], therefore, confirm that MINPP1 is a key endogenous enzyme in the generation of this important inositol phosphate. Interestingly, Ins(1,2,3)*P*_3_ metabolism is tightly controlled during cell cycle progression peaking when iron metabolism is upregulated [46,47]. Moreover, in the case of the MINPP1-associated human disease, there is a marked loss of intracellular iron [26], supporting a product of MINPP1 activity, like Ins(1,2,3)*P*_3_, regulating iron metabolism. 

## 5. Conclusions

Our experiments have shown that PtdIns(3,4,5)*P*_3_ is a novel target for hydrolysis by MINPP1. Thus, the ER confinement of MINPP1 allows the strategic separation of inositol polyphosphate metabolism from PI3K signaling, permitting important inositol phosphate metabolism related to the regulation of higher inositol phosphates to proceed without interfering with a critical mitogenic pathway (Figure 2F).

## Data Availability

The data that support the findings of this study are all provided within the body of the manuscript or the Supplementary Materials section.

## Supplementary Materials

The following supporting information can be downloaded at: https://www.mdpi.com/article/10.3390/biom13060885/s1, Figure S1: Localisation of GFP-tagged cyt-MINPP1; Figure S2: Lack of impact of cyt-MINPP1 expression on other inositol lipids.
