# Supplementary material for: Multiple Inositol Polyphosphate Phosphatase Compartmentalization Separates Inositol Phosphate Metabolism from Inositol Lipid Signaling
Source: Biomolecules. 2023 May 24;13(6):885. doi: 10.3390/biom13060885 (PMC10296752; doi:10.3390/biom13060885)
Supplement: Supplementary file 1 [file biomolecules-13-00885-s001.zip › biomolecules-2280314-supplementary.pdf]

Supplementary Materials

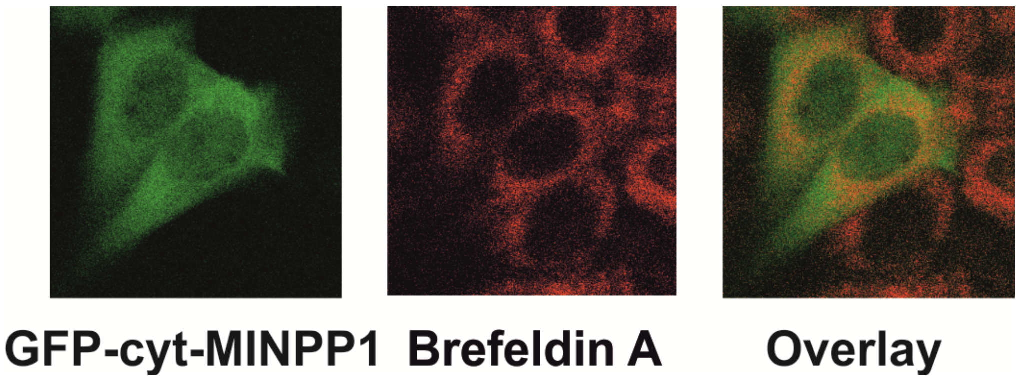

Supplemental Figure S1. Localisation of GFP-tagged cyt-MINPP1. HIT M2.2.2 cells were cotransfected with the combinations of GFP-cyt-MINPP1/ Brefeldin A, Bodipy 558/568 conjugate. The green colour is used as the digital pseudocolour for the fluorescence emitted from GFP and red is used as the digital pseudocolour for the fluorescence emitted from Brefeldin A, BODIPY 558/568 conjugate. The distribution of fluorescence was observed by laser scanning confocal microscopy. This experiment was repeated 2 more times.

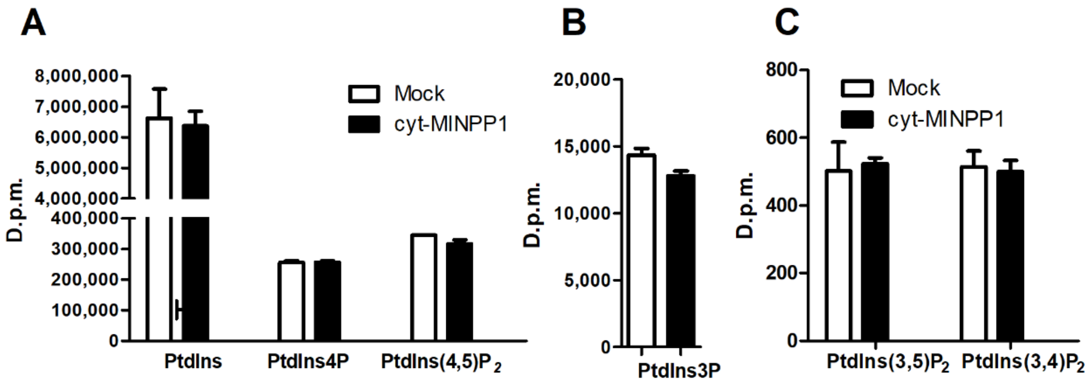

Supplemental Figure S2. Lack of impact of cyt-MINPP1 expression on other inositol lipids. Simultaneous with the determination of the deacylated PtdIns(3,4,5)P_3, using the same HPLC runs, we also determined the amounts of (**A**) the standard inositol lipids, again using their deacylated products. There was no significant impact of cyt-MIPP expression on these other inositol lipids. (**B**) There was a minor impact of the deacylation product of PtdIns3P which was barely statistically significant p = 0.0479, but (**C**) no impact on the minor PIP_2 isomers of PtdIns(3,5)P_2 and PtdIns(3,4)P_2. Data are averages of triplicate determinations in one experiment ± S.E.M. Two other experiments also in triplicate gave similar results.
